# Central bisectionectomy for hepatocellular carcinoma in a patient with indocyanine green excretory defect associated with reduced expression of the liver transporter

**DOI:** 10.1186/s40792-016-0216-8

**Published:** 2016-09-02

**Authors:** Shinya Imada, Tsuyoshi Kobayashi, Azusa Kitao, Osamu Matsui, Masakazu Hashimoto, Kentaro Ide, Kohei Ishiyama, Koji Arihiro, Hirotaka Tashiro, Hideki Ohdan

**Affiliations:** 1 Gastroenterological and Transplant Surgery, Applied Life Sciences, Institute of Biomedical and Health Sciences, Hiroshima University, 1-2-3 Kasumi, Minami-ku, Hiroshima 734-8551 Japan; 2 Department of Radiology, Kanazawa University Graduate School of Medical Science, Kanazawa, Japan; 3 Department of Pathology, Hiroshima University Hospital, Hiroshima, Japan

**Keywords:** Hepatocellular carcinoma, Indocyanine green excretory defect, Molecular transporter

## Abstract

**Background:**

Indocyanine green (ICG) excretory defect is a dye excretory disorder, and it is characterized by the selective impairment of plasma ICG clearance with normal liver histology. The pathophysiology involves selective loss of active transporters for ICG in the hepatic cell membrane. Several cases of hepatectomy in patients with ICG excretory defect have been reported, but the expression of hepatic transporters involved in ICG excretory defect has not been examined in these cases.

**Case presentation:**

An 81-year-old man who was hepatitis B and C virus negative was admitted to our hospital with a diagnosis of HCC. Abdominal computed tomography revealed an 8-cm-diameter tumor in hepatic segments 4 and 8. The retention rate of ICG at 15 min (ICGR_15_), which has been used to evaluate hepatic functional reserve, was markedly elevated (79.1 %), whereas other liver function test results, were normal. Therefore, we diagnosed the patient with HCC with an ICG excretory defect, and considered major hepatectomy. Central bisectionectomy was performed, and the postoperative course was uneventful. Microscopic examination of the resected specimen showed moderately differentiated HCC. Immunohistochemical staining and polymerase chain reaction analysis of a non-neoplastic site of the resected specimen showed very few expression of the organic anion-transporting polypeptide 1B3 (OATP1B3), which is usually expressed on the basolateral membrane of human hepatocytes and mediates the uptake of ICG.

**Conclusions:**

In this case, we present a case of hepatectomy for HCC in a patient with ICG excretory defect, which may be attributable to a congenital disorder of OATP1B3 expression; however, an ICG excretory defect did not seem to have any effect on the short-term prognosis after hepatectomy.

## Background

Hepatectomy is performed within the limits of hepatic functional reserve. For many years, the indocyanine green (ICG) test has been used to evaluate hepatic functional reserve; however, it is difficult to accurately assess hepatic functional reserve in the presence of an ICG excretory defect [1]. We encountered a rare case of hepatocellular carcinoma (HCC) with an ICG excretory defect treated with major hepatectomy on the basis of a normal hepatic uptake ratio on ^99m^Tc-diethylene triamine pentaacetic acid-galactosyl human serum albumin (GSA) liver scintigraphy and otherwise normal liver function test results. ICG excretory defect was first reported by Namihisa et al. in 1974, as a new type of dye excretory disorder [2]. It is characterized by the selective impairment of plasma ICG clearance with normal liver histology. The pathophysiology involves selective loss of active transporters for ICG in the hepatic cell membrane [3]. Recently, it has been reported that organic anion transporting polypeptides (OATPs; encoded by SLCOs) and Na^+^-taurocholate cotransporting polypeptides (NTCPs; encoded by SLC10A1), which are present in the basolateral membrane of human hepatocytes, are important transporters for the uptake of substances and xenobiotics, and are positively correlated with the transporters for ICG [4]. Here, we report the association between ICG excretory defect and the expression of OATPs and NTCPs. Although there are several previous reports of hepatectomy in patients with an ICG excretory defect, we believe that this is the first case of HCC with an excretory defect in which the expression of hepatic transporters was examined.

## Case presentation

**Fig. 1:**
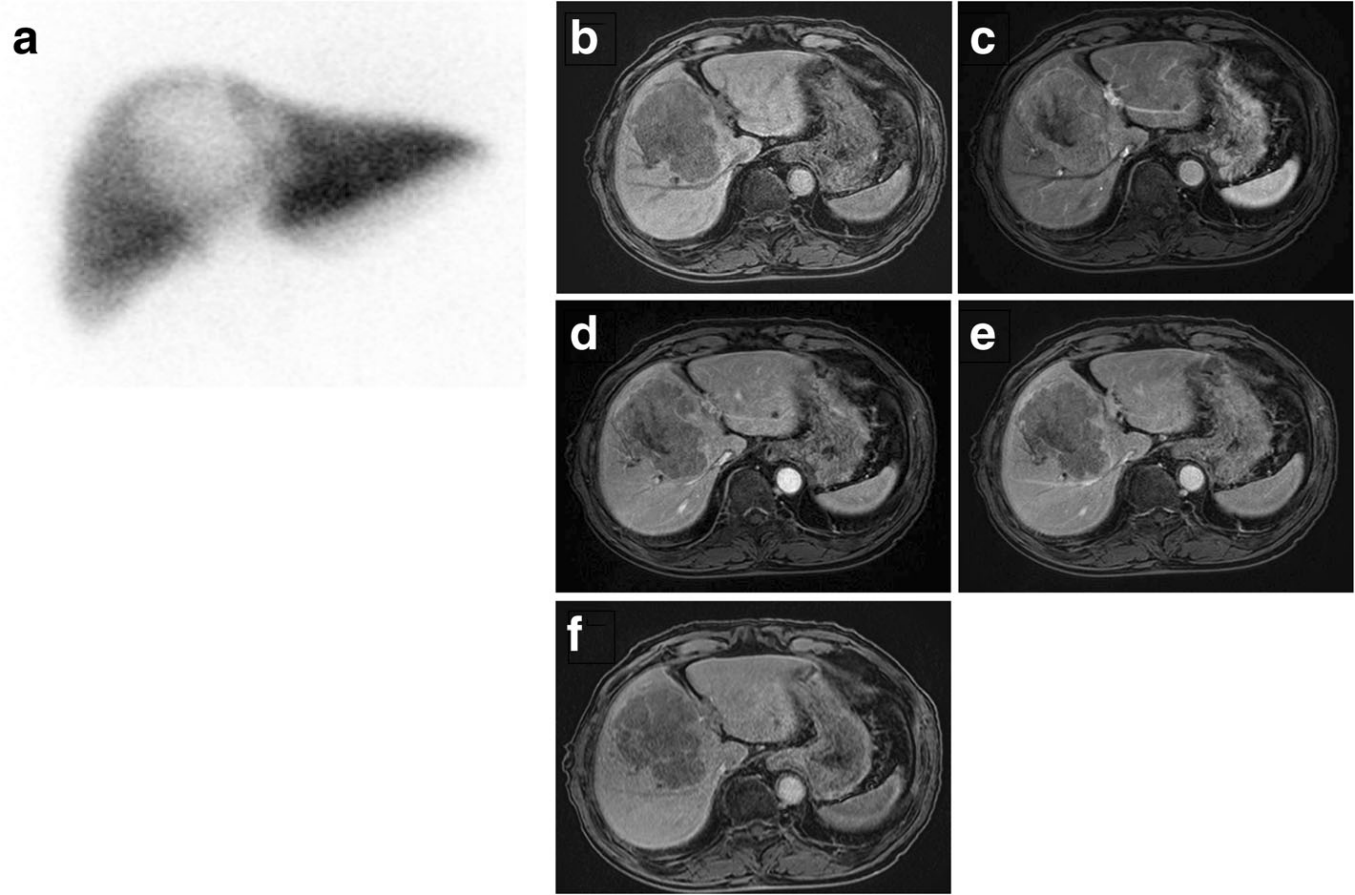
^99m^Tc-DTPA-galactosyl human serum albumin (GSA) liver scintigraphy showed normal accumulation of GSA in non-neoplastic lesion (**a**). Each gadolinium-ethoxybenzyl-diethylene triamine pentaacetic acid (Gd-EOB-DTPA)-enhanced magnetic resonance imaging (MRI) showed pre-contrast T1-weighted image (**b**), arterial dominant phase (**c**), portaldominant phase (**d**), transitional phase (**e**), and hepatobiliary phase (**f**). Hepatobiliary phase showed slight enhance with poor contrast enhancement to spleen or vessels in non-neoplastic site

An 81-year-old man who was hepatitis B and hepatitis C virus negative was admitted to our hospital with a diagnosis of HCC. He underwent preoperative examination for hepatectomy, including the ICG test. Physical examination indicated no tenderness in the abdomen or palpable mass. Blood tests showed slightly elevated levels of aspartate transaminase (65 IU/L; normal, <40 IU/L) and alanine transaminase (77 IU/L; normal, <40 IU/L), and markedly elevated levels of serum α-fetoprotein (125.5 ng/mL; normal, <6.5 ng/mL) and protein induced by vitamin K absence or antagonist II (5266 AU/mL; normal, <40 AU/mL). We performed ICG tests three times before hepatectomy; retention rates of ICG at 15 min (ICGR_15_) were markedly high, at 69.2, 58.5, and 79.1 %. All other laboratory data, including prothrombin activity (106 %; normal, >80 %), albumin level (3.9 g/dl; normal, >3.8 g/dl), and total bilirubin level (1.1 mg/dl; normal, <1.2 mg/dl), were normal, and the Child-Pugh score was A. Computed tomography (CT) revealed an 8-cm-diameter tumor in hepatic segments 4 and 8. Additionally, the tumor had heterogeneously high attenuation on CT during hepatic arteriography, and low attenuation on CT arterial portography. Angiography showed no mesosystemic or intrahepatic shunts. GSA liver scintigraphy showed reduced accumulation of GSA in segments 4 and 8 (Fig. 1a); the receptor index and index of blood clearance were normal, 0.91 (normal, >0.9) and 0.60 (normal, <0.61), respectively. In gadolinium-ethoxybenzyl-diethylene triamine pentaacetic acid (Gd-EOB-DTPA)-enhanced magnetic resonance imaging (MRI), the neoplastic site showed low signal intensity (Fig. 1b–e), and the non-neoplastic site was slightly enhanced on the hepatobiliary phase with poor contrast enhancement to spleen or vessels. The liver spleen contrast ratio of signal intensity was 1.25, which was rather low value compared to the previous report (Fig. 1f) [5]. CT volumetry estimated that the central bisection, which was the area of the expected resection, occupied 40 % of the total parenchymal volume. Based on the hepatic functional reserve of this patient, as indicated by GSA liver scintigraphy and liver function tests, central bisectionectomy was considered feasible. We decided to perform hepatectomy.

**Fig. 2:**
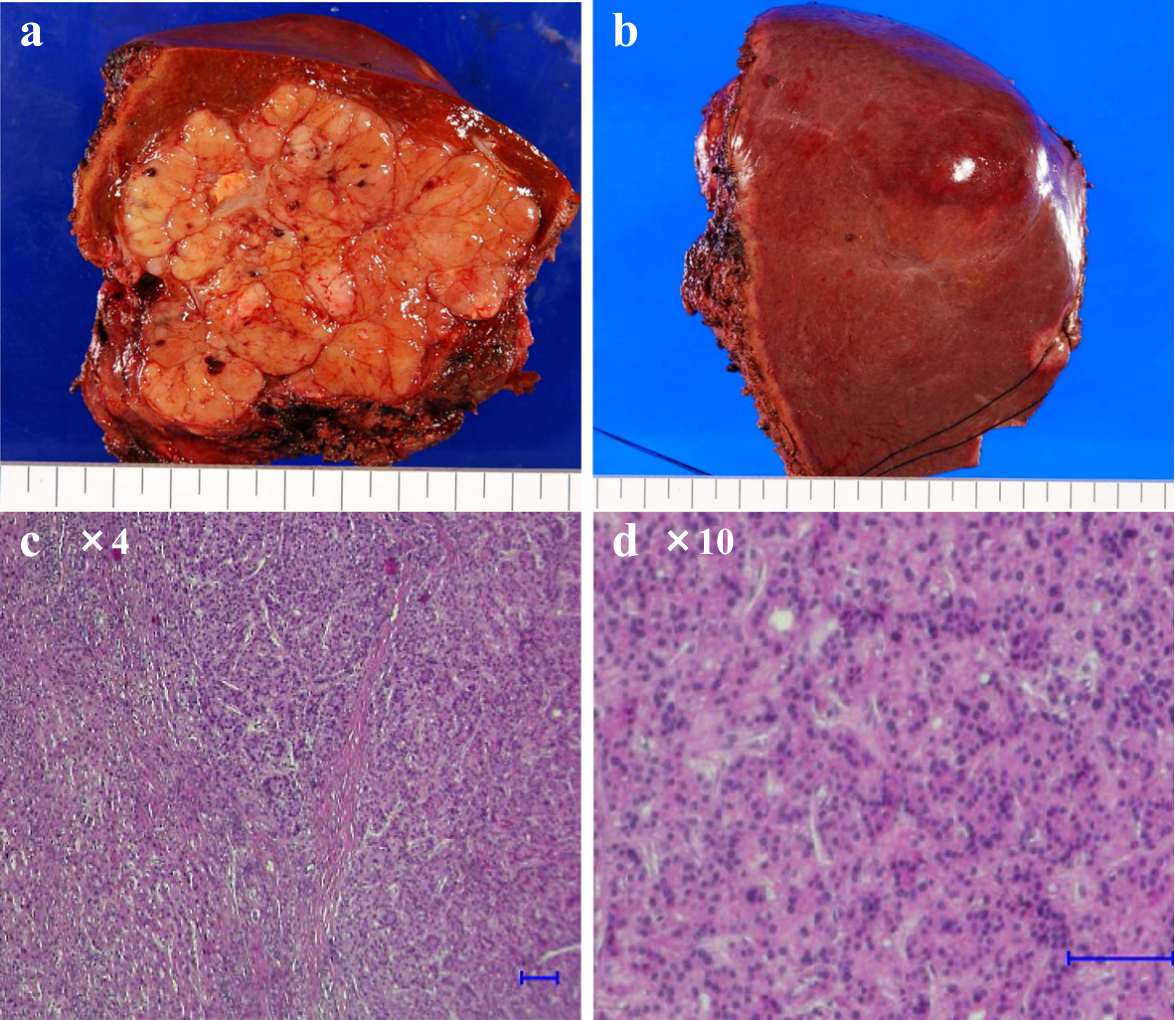
Gross appearance of the resected specimen (**a**, **b**). Pathological examination of the resected specimen showed moderately differentiated HCC and mild inflammation in neoplastic and non-neoplastic site (**c**, **d**)

**Fig. 3:**
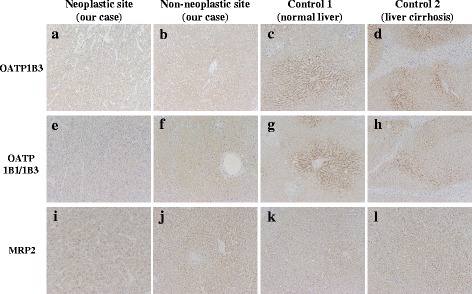
**a–l** Immunohistochemical staining of liver transporters in the resected specimens from our case and control cases

**Fig. 4:**
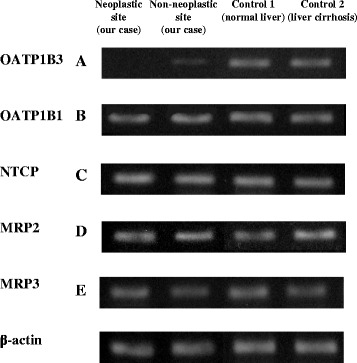
Reverse transcription polymerase chain reaction analysis of liver transporters in the resected specimens from our case and control cases (**a**–**e**) (normalization to β-actin expression)

Intraoperatively, the surface of the liver was smooth and the edge was sharp. There was no ascites, splenomegaly, mesosystemic shunt, or evidence of chronic hepatitis. Therefore, we performed central bisectionectomy without complications (Fig. 2a, b). The patient’s postoperative course was good, and he was discharged 3 weeks after hepatectomy. The ICGR_15_ 3 months after hepatectomy was still high at 62.9 %, although other blood test results, including levels of tumor markers, were normal. Pathological examination of the resected specimen revealed moderately differentiated HCC with mild chronic inflammation in the adjacent liver tissue (Fig. 2c, d). Immunohistochemical staining and reverse transcription polymerase chain reaction (RT-PCR) analysis of neoplastic and non-neoplastic sites of the resected specimens were examined in the same way as previous report [6]. Immunostaining was performed by using mouse monoclonal antibodies to OATP1B3 (NB100-74482, Novus Biologicals), OATP1B1 (ab15442, Abcam), and MRP2 (ab3373, Abcam). RT-PCR was done with following TaqMan® Gene Expression Assays obtained from Applied Biosystems (Warrington, England): OATP1B3 (encoded by SLCO1B3): Hs00251986_m1, OATP1B1 (encoded by SLCO1B1): Hs00272374_m1, NTCP (encoded by SLC10A1): Hs00161820_m1, MRP2 (encoded by ABCC2): Hs00166123_m1, MRP3 (encoded by ABCC3): Hs00978473_m1. Theses examinations revealed no or very few expressions of OATP1B3, which is localized to the basolateral membrane of human hepatocytes and mediates the uptake of agents such as ICG and Gd-EOB-DTPA (Fig. 3a–d and Fig. 4a). Expression of OATP1B1, another hepatic uptake transporter of the OATP family, in non-neoplastic and neoplastic sites was comparable with that in normal liver tissue on RT-PCR (Fig. 4b), but immunohistochemical staining indicated lower OATP1B1 expression than control (Fig. 3e–h). The non-neoplastic site’s expressions of Na^+^-taurocholate cotransporting polypeptide, a transporter that mediates uptake from the blood into hepatocyte, and multidrug resistance-associated protein 2 and 3 (MRP2 and MRP3), which mediate efflux from the hepatocyte into the bile and blood, were comparable with normal liver tissue (Fig. 3i–l and Fig. 4c–e).

### Discussion

Hepatectomy is the optimal curative treatment in patients with HCC. However, hepatectomy is associated with significant morbidity and mortality, especially in patients with underlying chronic liver disease [7]. ICG is a tricarbocyanine dye that was first introduced to measure blood flow, and later recognized as a useful compound for liver function testing [4]. Several studies have found the ICG clearance test to be the best test for selecting patients for hepatectomy [8]. Some investigators have used the ICG clearance test alone to exclude patients from hepatic resection if retention rates were not satisfactory [9], while others use the ICG retention rate to decide on the resection volume of the liver [10]. The ICG test is therefore well-established as a discriminating predictive test for the evaluation of hepatic functional reserve. However, in patients with an ICG excretory defect, the results of ICG tests become unreliable. An ICG excretory defect is indicated by a discrepancy between the ICG test findings and other conventional liver functional test results, and is characterized by selective impairment of plasma ICG clearance with normal liver histology. The pathophysiology involves the selective loss of active transporters for ICG in the hepatic cell membrane. As a result, ICG uptake into hepatocytes occurs by passive diffusion. The limited capacity of the intrahepatic transport of ICG may be an accessory factor in ICG excretory defect. With regard to hepatic transporters, de Graaf et al. reported that the transporter specificity of ^99m^Tc-mebrofenin and ICG partially overlap, as both compounds are transported by the hepatic uptake transporter OATP1B3. Furthermore, they reported that ^99m^Tc-mebrofenin was also taken up by OATP1B1, whereas ICG was additionally transported by NTCP [4]. Kitao et al. reported that the expression of both OATP1B3 and the export transporter MRP3 correlate significantly with the signal intensity of HCC in the hepatobiliary phase of Gd-EOB-DTPA-enhanced MRI [6]. In addition, they reported that decreased OATP1B3 expression during multistep hepatocarcinogenesis resulted in the decrease in enhancement ratio on Gd-EOB-DTPA-enhanced MRI [11]. These findings suggested that hepatic transporters for ICG, GSA, and Gd-EOB-DTPA are specific, but partially overlap.

In the present case, immunohistochemical staining and RT-PCR analysis of non-neoplastic and neoplastic sites revealed very few expression of the hepatic uptake transporter OATP1B3. It is possible that the deviation of the ICG test results from other liver function test results is attributable to a congenital disorder of OATP1B3.

Rotor syndrome (RS) should be considered in the differential diagnosis of diseases associated with high retention rates of ICG. Van de Steeg et al. reported that complete OATP1B1 and OATP1B3 deficiency caused human RS by interrupting conjugated bilirubin reuptake into the hepatocyte [12]. They analyzed the genome sequences of 11 RS index subjects from eight different families, and reported pathogenic mutations affecting both SLCO1B3 and SLCO1B1. These mutations resulted in disruption or annihilation of proper protein expression and function in each subject. We could not analyze genome sequence of our patient, although a pathogenic mutation of SLCO1B3 might disrupt OATP1B3 protein expression, resulting in the deviation of the ICG test results from other liver function test results.

**Fig. 5:**
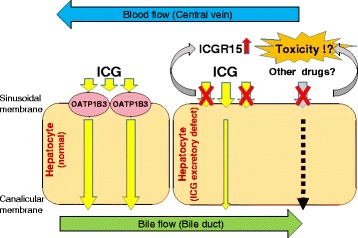
Schematic model of ICG excretory defect. The low expression of OATP1B3 induces high retention rate of ICG at 15 min (ICGR15), and have a possibility of other drug toxicities

Several cases of hepatectomy in patients with an ICG excretory defect have been reported [1, 3, 13, 14], and hepatectomy could be performed safely within the limits of hepatic functional reserve. It is certain that ICG excretory defect itself does not affect hepatic functional reserve from these results, but human OATP1B1 and OATP1B3 mediate the uptake of many drugs by the liver, and even polymorphisms of OATP1B1 and OATP1B3 that are associated with reduced activity can result in life-threatening drug toxicities [12]. Takane et al. reported that a specified genotype of uridine diphosphate glucuronosyltransferase 1A1 and SLCO1B1 caused life-threatening toxicity after irinotecan-based chemotherapy [15]. Patients who are candidates for surgical treatment will potentially receive neoadjuvant or adjuvant chemotherapy in the perioperative period. Although the patient in the present case did not develop severe liver dysfunction after hepatectomy, we have to consider the possibility of impaired expression of hepatocyte transporters, and exercise caution when using therapeutic drugs for patients with an ICG excretory defect (Fig. 5).

## Conclusions

In this case, we present a case of hepatectomy for HCC in a patient with ICG excretory defect, which may be attributable to a congenital disorder of OATP1B3 expression; however, an ICG excretory defect did not seem to have any effect on the short-term prognosis after hepatectomy.

## Abbreviations

CT: Computed tomography
Gd-EOB-DTPA: Gadolinium-ethoxybenzyl-diethylene triamine pentaacetic acid
GSA: ^99m^Tc-diethylene triamine pentaacetic acid-galactosyl human serum albumin
HCC: Hepatocellular carcinoma
ICG: Indocyanine green
ICGR_15_: Retention rate of ICG at 15 min
MRI: Magnetic resonance imaging
MRP: Multidrug-resistant protein
NTCP: Na^+^-taurocholate co-transporting polypeptide
OATP: Organic anion transporting polypeptide
